# Incidence and Mortality of Second Primary Cancers in Danish Patients With Retinoblastoma, 1943-2013

**DOI:** 10.1001/jamanetworkopen.2020.22126

**Published:** 2020-10-22

**Authors:** Pernille A. Gregersen, Maja H. Olsen, Steen F. Urbak, Mikkel Funding, Susanne O. Dalton, Jens Overgaard, Jan Alsner

**Affiliations:** 1 Department of Experimental Clinical Oncology, Aarhus University Hospital, Aarhus, Denmark; 2 Department of Clinical Genetics, Aarhus University Hospital, Aarhus, Denmark; 3 Center for Rare Disorders, Department of Pediatrics, Aarhus University Hospital, Aarhus, Denmark; 4 Unit of Survivorship, Danish Cancer Society Research Center, Copenhagen, Denmark; 5 Department of Ophthalmology, Aarhus University Hospital, Aarhus, Denmark; 6 Department of Clinical Oncology & Palliative Care, Zealand University Hospital, Naestved, Denmark

## Abstract

**Importance:**

In heritable retinoblastoma, there is a significantly increased risk of second primary cancers (SPCs). Improved knowledge about the incidence and influence of heritability and treatment is important during therapy for patients with retinoblastoma.

**Objective:**

To assess the incidence of SPC in patients diagnosed with retinoblastoma in Denmark from 1943 to 2013 with a focus on heritability and the association of external radiotherapy with mortality.

**Design, Setting, and Participants:**

In this retrospective cohort study, data were extracted from the Danish Ocular Oncology Group Database containing complete data on all patients diagnosed with retinoblastoma , and obtained from the Danish Cancer Registry, which includes information on all patients with cancer from 1943 to December 31, 2013. Data analysis was conducted from December 1, 2017, to October 1, 2019. Data on 323 patients were included.

**Exposures:**

Heritability and retinoblastoma treatment.

**Main Outcomes and Measures:**

Standardized incidence rate, excess absolute risk, cumulative incidence of SPC, and mortality from SPC. Association of heritability and treatment with outcomes was estimated.

**Results:**

Of the 323 patients included in the analysis, 181 were men (56%), 133 had heritable retinoblastoma (41%), and 190 had nonheritable retinoblastoma (59%). The median age at diagnosis of SPC was 32.4 (interquartile range, 15.4-43.9) years in patients with heritable retinoblastoma and 38.6 (interquartile range, 20.5-49.4) years in those with nonheritable retinoblastoma. Twenty-five SPCs were identified in patients with heritable retinoblastoma vs 14 in patients with nonheritable retinoblastoma. Standardized incidence rate (SIR) of SPC in patients with heritable retinoblastoma was 11.39 (95% CI, 7.37-16.81) with an excess absolute risk of 70 cases per 10 000 person-years; the highest SIRs were for sarcoma (181.13; 95% CI, 98.94-303.92) and malignant melanoma (26.78; 95% CI, 9.78-58.30). The SIR for SPC in patients with nonheritable retinoblastoma was 1.52 (95% CI, 0.81-2.60). The cumulative incidence of SPCs at age 60 years was significantly higher in patients with heritable retinoblastoma (51%) compared with those with nonheritable retinoblastoma (13%) (*P* < .001) (hazard ratio, 5.0; 95% CI, 2.5-10.3). No significant differences were identified in overall risk of SPC in patients with heritable retinoblastoma treated with 3 different modalities: external radiotherapy, plaque (but no external) radiotherapy, and enucleation only, but an increased proportion of sarcomas was noted in the irradiated field. Mortality due to SPC was also higher in survivors of heritable retinoblastoma compared with those with nonheritable retinoblastoma (cumulative mortality, 34% vs 12% at age 60 years; *P* = .03).

**Conclusions and Relevance:**

The findings of this study suggest that the incidence and mortality associated with SPC were significantly higher in patients with heritable retinoblastoma vs patients with nonheritable retinoblastoma. The largest increases in risk were noted for sarcoma and malignant melanoma. External radiotherapy did not appear to increase the risk. These findings are relevant when treating patients with retinoblastoma to manage the risk for SPC.

## Key Points

**Question:**

Are heritability and treatment associated with the incidence of second primary cancer in Danish retinoblastoma survivors?

**Findings:**

In this national cohort study of 323 patients in Denmark diagnosed with retinoblastoma, the incidence and mortality of second primary cancer were significantly higher in patients with heritable retinoblastoma vs patients with nonheritable retinoblastoma. The data did not show an increased risk in patients with heritable disease who were treated with external radiotherapy.

**Meaning:**

The findings of this study suggest that patients with a genetic predisposition to retinoblastoma may be at greater greatest risk for developing second primary cancer later in life.

## Teaser

This cohort study examines the incidence of and mortality associated with second primary cancers that develop after diagnosis of retinoblastoma in Danish patients.

## Introduction

Retinoblastoma is a rare intraocular childhood cancer caused by a pathogenic variant (formerly termed a mutation) in both *RB1* (OMIM 614041) alleles.^1^ In patients with heritable retinoblastoma, the patient has a germ-line *RB1* pathogenic variant, and a new sporadic pathogenic variant in the other *RB1* gene initiates tumorigenesis.^2^ The *RB1* germ-line variant can be inherited from an affected parent, but is most often a de novo variant.^3^ In *RB1* mosaicism, the *RB1* variant is present in only a fraction of cells and tissues.^4,5^

Nonheritable retinoblastoma presents as unilateral disease (mean age at diagnosis, 24 months), and heritable retinoblastoma primarily presents as bilateral and/or multifocal disease (mean age at diagnosis, 15 months).^6^ Up to 14% of unilaterally affected patients with retinoblastoma without a family history carry an *RB1* germ-line variant.^1,6^ Patients with heritable retinoblastoma have a lifelong increased risk of developing second primary cancers (SPCs).^7,8,9,10,11,12,13,14,15,16,17,18,19,20,21,22^ This risk may be increased further in patients treated with radiotherapy.^10,11,12,19,22^

We describe the incidence and mortality of SPC in 323 patients with retinoblastoma in Denmark from 1943 to 2013. To our knowledge, this study is unique in the completeness of the nationwide data collected and the long-term follow-up.^9,23,24,25,26^

## Methods

### Patients

Denmark’s population is 5.8 million, and retinoblastoma treatment has been centralized in Aarhus since 1963. Data for this study were extracted from the Danish Ocular Oncology Group Database, which contains complete data on all Danish patients with retinoblastoma diagnosed since the establishment of the Danish Cancer Registry in 1943. The database holds the oldest national consecutive population of patients with retinoblastoma, and follow-up is complete through December 31, 2013. Selected data have been published previously^9,24,25,26,27,28^; the most recent publication describes the cohort in detail.^29^According to Danish regulations, approval of the study by an ethics committee and informed consent from study participants was not needed. This study followed the Strengthening the Reporting of Observational Studies in Epidemiology (STROBE) reporting guideline for cohort studies.

For the past decade, most patients with newly diagnosed retinoblastoma have undergone *RB1* testing, and many older patients have been tested as a result of implementing genetic testing or another reason.^29^ Because the study cohort contains both genetically tested and untested patients, heritable retinoblastoma was defined by bilateral or multifocal disease, familial presentation, and/or identification of an *RB1* variant. Nonheritable retinoblastoma was defined by unilateral, unifocal, and sporadic disease.

Data on SPCs were extracted from the Danish Cancer Registry, which was the world’s first population-based national cancer registry containing systematically collected information on all incident cases of cancer in Denmark.^30,31^ All registered cancers were included; benign tumors, including nonmelanoma skin cancer and in situ carcinomas, were excluded.

All Danish citizens have free access to medical care and a unique personal identification number assigned by the National Central Population Registry, allowing linkage between registers and databases at an individual level.^30^

The study included all 323 patients with retinoblastoma diagnosed in Denmark between 1943 and 2013 and followed up until December 31, 2014. Three additional individuals, either unaffected *RB1* variant carriers and/or diagnosed with retinoma (retinocytoma) or retinal scars, were not included.

### Statistical Analysis

All patients had follow-up for the new primary cancer from the date of retinoblastoma diagnosis until the date of emigration, death, or end of follow-up on December 31, 2014, whichever came first. Median years of follow-up and age at follow-up were calculated using reverse Kaplan-Meier analysis. Treatment data were available for all patients. Based on the person-years at risk, standardized incidence rates (SIRs) of new primary cancers and 95% CIs were calculated from the observed and expected numbers of cancers, with the expected number based on national incidence rates by sex, year of diagnosis (in 5-year intervals), and age at diagnosis (in 5-year intervals). The observed to expected ratio is interpreted as a measure of the risk of developing a new primary cancer after the retinoblastoma diagnosis, assuming that this risk is equal to the sex-, age-, and time-specific risk of developing cancer in the general population. The excess absolute risk per 10 000 person-years was calculated as observed − expected / person-years × 10 000. The cancer types were grouped into main categories according to a modification of the *International Statistical Classification of Diseases, Tenth Revision*, which includes a translation of the *International Classification of Diseases, Seventh Revision* codes for cancer diagnosed before 1977.^32^ Sarcoma was defined as described in the eTable in the Supplement. No similar methods exist for classification of carcinomas in the Danish Cancer Registry. For the SIR and excess absolute risk analysis, 1 case of second cancer diagnosed before the diagnosis of retinoblastoma was excluded. The difference in median age at diagnosis of SPC was tested by Wilcoxon rank-sum analysis. Cumulative incidence rates were calculated with competing risk adjusting for death and compared by the Wald test. Cox regression proportional hazards analyses were used to estimate hazard ratios with 95% CIs. Cumulative mortality rates for specific causes of death were calculated with competing risk adjusting for other causes of death and compared by the Wald test. The Fisher exact test was used in the analysis of contingency tables. Propensity score matching was used to compare the risk of SPC by heritable vs nonheritable categorization using sex, age at retinoblastoma diagnosis, and year of retinoblastoma diagnosis (Table 1) as covariates. *P* values were 2-sided, and *P* < .05 was considered statistically significant for all comparisons. All analyses were performed using SAS, version 9.4 (SAS Institute Inc) and Stata, version 15.1 (StataCorp). Data analysis was conducted from December 1, 2017, to October 1, 2019.

**Table 1.** Characteristics of Patients With Retinoblastoma Diagnosed From 1943 to 2013

Characteristic	Patients, No. (%)		
	Total (N = 323)	Heritable (n = 133)^a^	Nonheritable (n = 190)^b^
**Patient characteristics**			
Laterality			
Unilateral	206 (64)	16 (12)	190 (100)
Bilateral	117 (36)	117 (88)	0
Positive family history^c^			
No	274 (85)	84 (63)	190 (100)
Yes	49 (15)	49 (37)	0
Sex			
Female	142 (44)	58 (44)	84 (44)
Male	181 (56)	75 (56)	106 (56)
Age at retinoblastoma diagnosis, y			
<1	128 (40)	86 (65)	42 (22)
1	84 (26)	34 (26)	50 (26)
2	60 (19)	12 (9)	48 (25)
≥3	51 (16)	1 (1)	50 (26)
Year of retinoblastoma diagnosis			
1943-1966	101 (31)	35 (26)	66 (35)
1967-1992	117 (36)	53 (40)	64 (34)
1993-2013	105 (33)	45 (34)	60 (32)
**Treatment combinations**^d^			
Radiotherapy			
Alone	29 (9)	23 (17)	6 (3)
With focal therapy	2 (1)	2 (2)	0
With chemotherapy	4 (1)	4 (3)	0
With chemotherapy and focal therapy	2 (1)	2 (2)	0
With chemotherapy and enucleation	9 (3)	8 (6)	1 (1)
With chemotherapy, enucleation, and focal therapy	4 (1)	3 (2)	1 (1)
With enucleation	91 (28)	73 (55)	18 (9)
With enucleation and focal therapy	2 (1)	2 (2)	0
Chemotherapy			
Alone	1 (0)	1 (1)	0
With focal therapy	1 (0)	1 (1)	0
With enucleation	7 (2)	2 (2)	5 (3)
With enucleation and focal therapy	0	0	0
Enucleation alone	171 (53)	12 (9)	159 (84)
**Treatment groups**			
Radiotherapy ± focal therapy	31 (10)	25 (19)	6 (3)
Radiotherapy with chemotherapy	6 (2)	6 (5)	0
Radiotherapy, chemotherapy, and enucleation	13 (4)	11 (8)	2 (1)
Radiotherapy with enucleation	93 (29)	75 (56)	18 (9)
Chemotherapy ± focal therapy	2 (1)	2 (2)	0
Chemotherapy with enucleation	7 (2)	2 (2)	5 (3)
Enucleation alone	171 (53)	12 (9)	159 (84)
**Radiotherapy combinations**			
External kV alone	22 (7)	17 (13)	5 (3)
External kV with plaque cobalt 60	2 (1)	2 (2)	0
External kV with plaque ruthenium	0	0	0
External MV alone	57 (18)	46 (35)	11 (6)
External MV with plaque cobalt 60	2 (1)	2 (2)	0
External MV with plaque ruthenium	8 (2)	8 (6)	0
External MV and plaque ruthenium with protons	1(0)	1 (1)	0
Plaque cobalt 60 alone	33 (10)	33 (25)	0
Plaque ruthenium alone	18 (6)	8 (6)	10 (5)
None	180 (56)	16 (12)	164 (86)
**Radiotherapy groups**			
External kV/MV alone	79 (24)	63 (47)	16 (8)
External kV/MV with plaque	12 (4)	12 (9)	0
External kV/MV, plaque, and protons	1 (0)	1 (1)	0
Plaque alone	51 (16)	41 (31)	10 (5)
None	180 (56)	16 (12)	164 (86)

^a^ Defined as bilateral or multifocal disease, positive family history, and/or *RB1* variant identified.

^b^ Defined as sporadic, unilateral, and unifocal disease.

^c^ Familial cases inherited the disease from 1 parent.

^d^ Focal therapy included cryotherapy or laser transpupillary thermotherapy.

## Results

### Cohort

In this retrospective national cohort study, retinoblastoma was diagnosed in a total of 323 patients between 1943 and December 31, 2013: 142 women (44%) and 181 men (56%) (Table 1). Of these, 133 patients (41%) had heritable retinoblastoma and 190 patients (59%) had nonheritable retinoblastoma. The proportion of patients with heritable retinoblastoma was lower for patients diagnosed through 1966 (35/101 [35%]) compared with those diagnosed after (98/222 [44%]), and the proportion of familial cases also increased after 1966 (10/101 [10%] vs 39/222 [18%]). Forty-eight patients died and 9 emigrated; no other patients were lost to follow-up. Median years of follow-up were 24.5 (95% CI, 20.2-30.3) years for patients with heritable retinoblastoma and 32.0 (95% CI, 25.1-37.2) years for those with nonheritable retinoblastoma, and median age at follow-up was 25.9 (95% CI, 20.8-31.4) years for patients with heritable retinoblastoma and 33.7 (95% CI, 27.8-40.3) years for those with nonheritable retinoblastoma.

### Treatment

The Danish guidelines for the treatment of retinoblastoma have changed since 1943. Initially, treatment included enucleation or orthovoltage radiotherapy, and the focus was primarily to secure survival. Treatment evolved to save vision in patients with bilateral retinoblastoma by administering radiotherapy to the least affected eye. In 1963, diagnosis, treatment, and follow-up became centralized in Aarhus and, over time, strategies were developed to preserve vision and improve cosmetic outcome. In 1964, orthovoltage irradiation was replaced by high-voltage external radiotherapy, initially with cobalt 60 irradiation and beginning in 1981, with 4 to 6 MV linear accelerator. Plaque therapy with cobalt 60 was used from 1957 to 1980, and ruthenium plaques were introduced in 1989. Systemic chemotherapy was implemented in 2003, cryotherapy was introduced in 2005, and laser transpupillary thermotherapy was instituted in 2012. Table 1 lists the combinations of therapy.

### SIRs and Excess Absolute Risk of SPC

Thirty-seven patients with retinoblastoma had a total of 39 SPCs diagnosed. Table 2 summarizes the cases according to morphologic characteristics (carcinoma, malignant melanoma, sarcoma, or central nervous system), with median age at diagnosis, heritability, use of external radiotherapy, and whether a case was considered potentially radiation induced (ie, located inside the irradiation field). One patient developed an SPC before the retinoblastoma diagnosis, and this case was not included in SIR and excess absolute risk analysis. Thus, 38 cases were included in the SIR and excess absolute risk analysis.

**Table 2.** SPC in Patients With Retinoblastoma Diagnosed From 1943-2013

SPC^a^	All patients (N = 323)		Heritable (n = 133)^b^								Nonheritable (n = 190)^c^							
	Cases, No.	Age at diagnosis, median (IQR), y	Observed, No.	Expected, No.	SIR (95% CI)^d^	Excess absolute risk^e^	External radiotherapy (n = 76), No.		No external radiotherapy (n = 57), No.		Observed, No.	Expected, No.	SIR (95% CI)^d^	Excess absolute risk^e^	External radiotherapy (n = 16), No.		No external radiotherapy (n = 174), No.	
							Cases	In RT field	Cases	In RT field					Cases	In RT field	Cases	In RT field
All	39	34 (15-47)	25	2.20	11.39 (7.37-16.81)	70.26	13	6	12	1	13	8.55	1.52 (0.81-2.60)	7.47	0	0	14	3
Carcinoma	8	53 (45-56)	3	NA	NA	NA	1	0	2	0	5	NA	NA	NA	0	0	5	0
Malignant melanoma	9	30 (24-39)	6	0.22	26.78 (9.78-58.30)	17.81	2	0	4	1	3	0.64	4.68 (0.94-13.67)	3.96	0	0	3	0
Sarcoma	18	24 (13-45)	14	0.08	181.13 (98.94-303.92)	42.90	8	5	6	0	4	0.18	22.68 (6.10-58.07)	6.41	0	0	4	1
CNS	4	4 (2-20)	2	0.27	7.43 (0.83-26.84)	5.33	2	1	0	0	1	0.66	1.52 (0.02-8.45)	0.57	0	0	2	2

Abbreviations: CNS, central nervous system; IQR, interquartile range; NA, not analyzed; RT, radiotherapy; SIR, standardized incidence rate; SPC, second primary cancer.

^a^ Three patients were not included in the cumulative incidence analysis: 1 carcinoma and 1 malignant melanoma were diagnosed as third primary cancers in 2 patients, and 1 sarcoma was diagnosed after age 60 years. One nonheritable CNS case was excluded from SIR and excess absolute risk analysis because it was diagnosed before retinoblastoma was identified.

^b^ Defined as bilateral or multifocal disease, positive family history, and/or *RB1* variant identified.

^c^ Defined as sporadic, unilateral, and unifocal disease.

^d^ Standardized incidence rate = observed / expected.

^e^ Excess absolute risk = observed – expected / person-years × 10 000.

There were no substantial differences between men and women, except that all 3 patients with heritable retinoblastoma who developed a carcinoma were women (2 breast and 1 ovarian cancer). In 5 patients with nonheritable retinoblastoma who developed a carcinoma, 3 were women (1 lung, 1 breast, and 1 cervical cancer) and the remaining 2 patients were men (1 lung and 1 thyroid cancer).

Table 2 also summarizes the cases for the SIR and excess absolute risk analysis by heritability. In patients with heritable retinoblastoma, 25 cases of SPC were observed. In the nonheritable retinoblastoma group, 13 cases were observed. Of the 25 cases in patients with heritable retinoblastoma, 14 cases (56%) were sarcoma, 6 cases (24%) were malignant melanoma, 3 cases (12%) were carcinoma, and 2 cases (8%) were central nervous system tumors. Of the 13 cases in the nonheritable retinoblastoma group, 5 cases (38%) were carcinoma, 4 cases (31%) were sarcoma, 3 cases (23%) were malignant melanoma, and 1 case (8%) was a central nervous system tumor. The median age at diagnosis of SPC did not differ significantly in patients with heritable retinoblastoma (32.4 years; interquartile range, 15.4-43.9) vs nonheritable retinoblastoma (38.6 years; interquartile range, 20.5-49.4) (*P* = .68).

In patients with heritable retinoblastoma, the observed number of SPCs was significantly higher than expected: SIR, 11.39 (95% CI, 7.37-16.81), excess absolute risk was 70.26 cases per 10 000 person years (Table 2). In particular, the risks were significantly higher for sarcoma (SIR, 181.13; 95% CI, 98.94-303.92) and malignant melanoma (SIR, 26.78; 95% CI, 9.78-58.30).

In contrast, the overall risk of any SPC was not significantly higher in patients with nonheritable retinoblastoma (SIR, 1.52; 95% CI, 0.81-2.60); only the risk of sarcoma was found to be higher (SIR, 22.68; 95% CI, 6.10-58.07). Propensity score matching, using sex, age at retinoblastoma diagnosis, and year of retinoblastoma diagnosis as covariates, showed that the risk of SPC was higher in patients with heritable vs nonheritable retinoblastoma (*P* = .01).

### Cumulative Incidence

**Figure 1.**
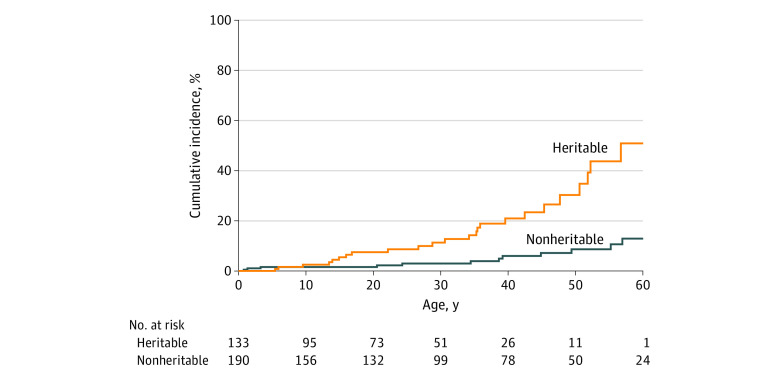
Cumulative Incidence of Second Primary Cancers at Age 60 Years Grouped by Heritability Wald test at age 60 years, *P* < .001; hazard ratio, 5.0 (95% CI, 2.5-10.3).

The cumulative incidence of SPCs at age 60 years grouped by heritability is shown in Figure 1. Cases include 36 SPCs in the 39 patients reported in Table 2, excluding 2 third primary cancers occurring after the first SPC in 2 patients and 1 third primary cancer occurring after the age of 60 years. The cumulative incidence in patients with heritable retinoblastoma (51%) was significantly higher than in those with nonheritable retinoblastoma (13%) (*P* < .001), with a hazard ratio of 5.0 (95% CI, 2.5-10.3).

### Risk With External Radiotherapy

**Figure 2.**
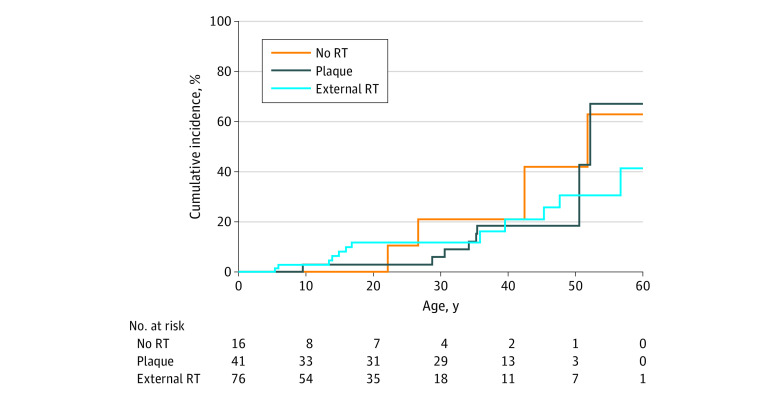
Cumulative Incidence of Second Primary Cancers in Patients With Heritable Retinoblastoma Treated With Different Modalities RT indicates radiotherapy.

The cumulative incidence of SPCs in patients with heritable retinoblastoma treated with different modalities is shown in Figure 2. Three groups of patients were included: 1 group treated with external radiotherapy as part of the treatment, 1 group treated with plaque (but not external) radiotherapy as part of the treatment, and 1 group treated without radiotherapy. Chemotherapy was given to 12 patients in the external radiotherapy group (16%), 5 in those who received plaque alone group (12%), and 4 who did not receive radiotherapy (25%) (*P* = .49). Of the 16 patients treated without radiotherapy, 14 were treated with enucleation (2 of them also received chemotherapy), 1 with chemotherapy alone, and 1 with chemotherapy and focal therapy (laser transpupillary thermotherapy). The cumulative incidence of SPC at age 60 years was 41% (95% CI, 19%-63%) for external radiotherapy, 67% (95% CI, 17%-91%) for plaque alone, and 63% (95% CI, 31%-83%) for no radiotherapy, showing no significant difference between the groups (Figure 2). Similarly, the 3 groups had comparable SIR values: external radiotherapy, 10.96 (95% CI, 5.83-18.74); plaque alone, 9.99 (95% CI, 4.30-19.69); and no radiotherapy, 19.19 (95% CI, 5.16-49.14). The combined SIR for no external radiotherapy was 11.90 (95% CI, 6.14-20.78).

Although there was no overall increased risk of SPCs in patients with heritable retinoblastoma treated with external radiotherapy, there was an increased proportion of sarcomas located in the irradiated field. In the 76 patients with heritable retinoblastoma treated with external radiotherapy, 5 of 8 cases of sarcoma were observed in the irradiated field, whereas 0 of 6 cases were observed in the similar area in the 57 patients with heritable retinoblastoma treated without external radiotherapy (*P* = .03) (Table 2).

### Mortality

**Figure 3.**
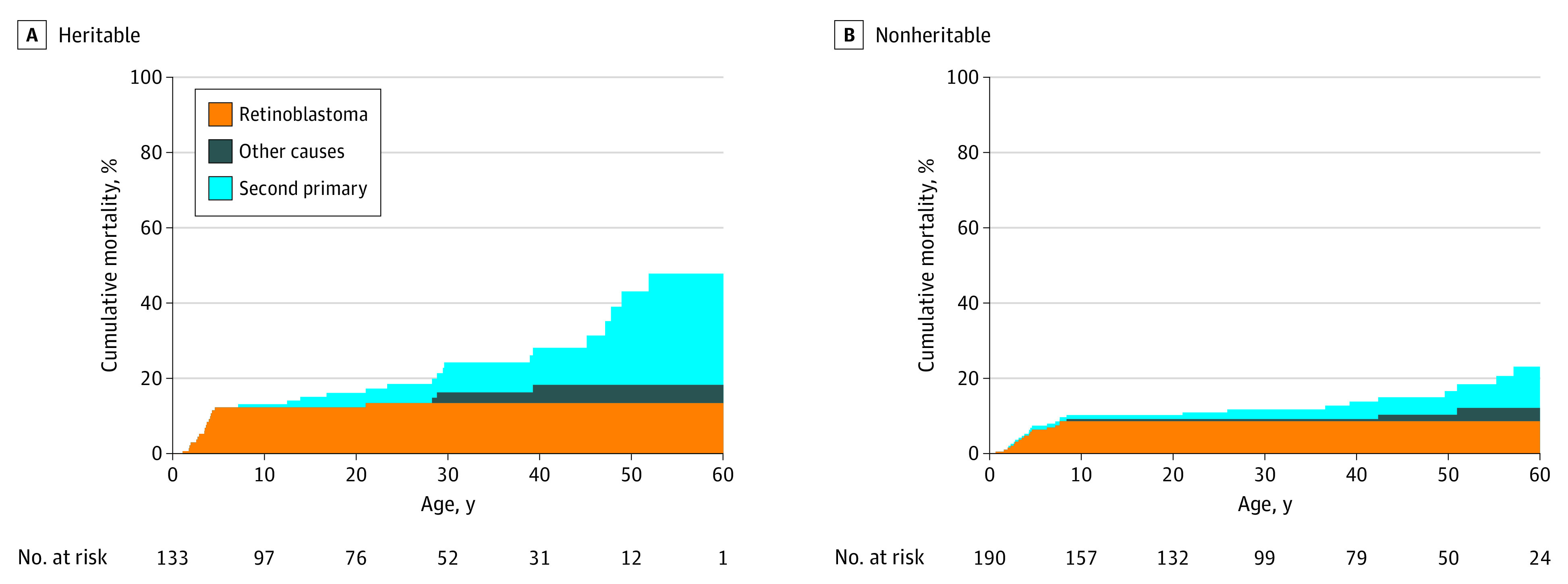
Cumulative Mortality Rate by Heritability

The cumulative mortality rate as death from retinoblastoma, SPC, and other known causes is shown in Figure 3. The risk of death from retinoblastoma and other known causes was similar in patients with heritable (Figure 3A) and nonheritable disease (Figure 3B). The risk of death from retinoblastoma decreased significantly since 1943.^29^ The risk of death from SPC was studied in the 290 patients surviving retinoblastoma: among the 116 with heritable retinoblastoma, 13 patients (11%) died of SPC and 3 patients (3%) died of other known causes; among 174 patients with nonheritable retinoblastoma, 9 patients (5%) died of SPC and 3 patients (2%) died of other known causes. The overall cumulative mortality rate was 48% for patients with heritable retinoblastoma and 23% for patients with nonheritable retinoblastoma. Analyzing data on patients surviving retinoblastoma, the cumulative mortality rate from SPC at age 60 years was 34% among those with heritable retinoblastoma and 12% among those with nonheritable retinoblastoma (*P* = .03).

## Discussion

In this study, patients with heritable retinoblastoma had a significantly increased risk for SPCs (SIR, 11.39; 95% CI, 7.37-16.81), while patients with nonheritable retinoblastoma did not (SIR, 1.52; 95% CI, 0.81-2.60). The cumulative incidence of SPC at age 60 years was also significantly higher in patients with heritable retinoblastoma (51%) vs patients with nonheritable retinoblastoma (13%) (hazard ratio, 5.0; 95% CI, 2.5-10.3). The types of SPC differed between the 2 groups: sarcoma and malignant melanoma were the most common SPCs in patients with heritable retinoblastoma, and carcinoma was the most common type in patients with nonheritable retinoblastoma.

We did not observe any significant difference in cumulative incidence or SIR when patients with heritable retinoblastoma were stratified by treatment modality. Patients who received external radiotherapy as part of the treatment had a cumulative incidence of 41% at age 60 years and an SIR of 10.96 (95% CI, 5.83-18.74). Patients who received plaque alone radiotherapy as part of the treatment had a cumulative incidence of 67% and an SIR of 9.99 (95% CI, 4.30-19.69). Patients treated without radiotherapy had a cumulative incidence of 63% and an SIR of 19.19 (95% CI, 5.16-49.14). Furthermore, we found that the cumulative mortality rate from SPCs was significantly higher for patients with heritable retinoblastoma compared with patients with nonheritable retinoblastoma.

The proportion of patients with heritable retinoblastoma was lower for patients diagnosed in 1966 (35%) vs after 1966 (44%). This difference could reflect the lower frequency of genetic tests in patients diagnosed early in the period,^29^ resulting in a higher risk of misclassifying sporadic unilateral heritable cases as nonheritable. Also, the proportion of familial cases increased over time, probably reflecting improved survival rates following changes in treatment guidelines.

The increased risk of SPCs in heritable retinoblastoma has been well known for many decades, and the increased incidence of SPCs in our complete nationwide cohort of patients with heritable retinoblastoma supports the findings of previous studies.^7,8,9,10,11,12,13,14,15,16,17,18,19,20,21,22^ The risk is caused by constitutional *RB1* pathogenic variant, compromising tumor suppressor function.

In addition, a higher incidence of lipoma and pineoblastoma has been reported in heritable retinoblastoma compared with nonheritable retinoblastoma.^11,33^ Lipoma was not included in the present study. Pineoblastoma is histologically identical to retinoblastoma and is termed *trilateral retinoblastoma*,^34^ ie, not classified as an SPC, and none were observed in this cohort.

Our findings for patients with heritable retinoblastoma (cumulative incidence of SPC of 51% at age 60 years, in particular, increased risk of sarcoma and malignant melanoma) are comparable to 4 other large studies with long term follow-up. In a study of British survivors of retinoblastoma born between 1873 and 1950, the cumulative cancer incidence for 144 patients with heritable retinoblastoma was 69% at age 84 years.^14^ A US study found that the cumulative incidence of an SPC among 963 heritable retinoblastoma survivors (diagnosed from 1914 to 1984) was 36% at 50 years after diagnosis, with sarcoma, melanoma, and cancer of the brain and nasal cavity being the most common types of cancer.^15^ A study from the Netherlands of patients diagnosed from 1945 to 2005 found a cumulative incidence of 28% among 298 heritable retinoblastoma survivors for any second cancer 40 years after diagnosis of retinoblastoma, with sarcoma, malignant melanoma, and epithelial cancers (bladder cancer and lung cancer) being the most frequent.^16^ In addition, a German study reported an SIR of 179 for sarcoma among 648 patients with heritable retinoblastoma treated from 1940 to 2008, which is similar to the SIR of 181.1 found in our study.^21^

In apparent contrast to previous studies on associations between treatment of retinoblastoma and incidence of or mortality due to SPC,^10,11,12,15,16,19,20,21,22,35^ our data did not show an increased overall risk in the patients with heritable retinoblastoma treated with external radiotherapy. However, we observed that a higher proportion of sarcomas was found inside the irradiated field in heritable retinoblastoma treated with external radiotherapy compared with no external radiotherapy. Other studies have reported that chemotherapy is an independent risk factor for SPCs among retinoblastoma survivors, particularly sarcoma, but apparently less so for malignant melanoma and epithelial tumors.^19,21^ Chemotherapy has been used in Denmark only since 2003 on a limited number of patients equally distributed between radiotherapy treatments and is therefore of minimal importance for the results presented here.

Consistent with previous studies,^10,11,14,20,22^ we found that survivors of heritable retinoblastoma had a higher overall mortality rate than survivors of nonheritable retinoblastoma and that the increased mortality may be associated solely with SPC. We found a cumulative mortality rate for patients with heritable retinoblastoma of 48% at age 60 years, and 2 large studies have reported mortality rates for patients with heritable retinoblastoma of 21% at 40 years^20^ and 75% at 70 years^22^ after diagnosis.

Surveillance for SPCs seems feasible, considering the significantly increased risk in survivors of heritable retinoblastoma. To our knowledge, no specific screening protocols have been published. However, annual oncologic workup is now routine at several retinoblastoma centers, and whole-body magnetic resonance imaging at regular intervals has been suggested.^6^ From the results of a recent pilot study, whole-body magnetic resonance imaging appeared to have limited sensitivity (67%) in detecting SPCs in survivors of heritable retinoblastoma, and any association with mortality, as well as whether the benefits outweigh the potential risks (eg, emotional stress and cost), are still unclear.^36^

### Strengths and Limitations

To our knowledge, this study is unique for its completeness of nationwide collected data, the extent of long-term follow-up, and uniformity in retinoblastoma assessment and follow-up owing to centralized management. The major limitation of the study was the small number of patients, and not all patients underwent genetic testing. Up to 14% of unilateral sporadic affected patients may carry an *RB1* pathogenic variant.^1,6^ Some patients classified as having nonheritable retinoblastoma were likely misclassified, causing underestimation of SPC incidence in those with heritable retinoblastoma and overestimation of those with nonheritable retinoblastoma. Although an increased risk of sarcoma (4 cases) among patients with nonheritable retinoblastoma was observed, 2 of the 4 patients were diagnosed with retinoblastoma in 1946 at the ages of 1 month and 10 months. The median age of diagnosis in the entire cohort was 8 months for patients with heritable and 2 years for those with nonheritable retinoblastoma, and it is possible that these 2 patients could have had heritable disease. In addition, the exclusion of 3 *RB1* variant carriers with no diagnosis of retinoblastoma could have affected data.

## Conclusions

The findings of this study suggest that patients with heritable retinoblastoma have a high risk of developing an SPC, primarily sarcoma and malignant melanoma. Patients with heritable retinoblastoma treated with or without external beam irradiation showed the same overall increased risk, but an increased proportion of sarcomas was observed inside the radiotherapy field in patients with heritable retinoblastoma treated with external beam irradiation.

## Notes

Supplement. **eTable.** Definition of Sarcomas for Calculating Standardized Incidence Ratios
