# Supplementary material for: Incidence and Mortality of Second Primary Cancers in Danish Patients With Retinoblastoma, 1943-2013
Source: JAMA Netw Open. 2020 Oct 22;3(10):e2022126. doi: 10.1001/jamanetworkopen.2020.22126 (PMC7582127; doi:10.1001/jamanetworkopen.2020.22126)
Supplement: Supplement. — eTable. Definition of Sarcomas for Calculating Standardized Incidence Ratios [file jamanetwopen-e2022126-s001.pdf]

Supplementary Online Content

Gregersen PA, Olsen MH, Urbak SF, et al. Incidence and mortality of second primary cancers in Danish patients with retinoblastoma, 1943-2013. *JAMA Netw Open*. 2020;3(10):e2022126. doi:10.1001/jamanetworkopen.2020.22126

eTable. Definition of Sarcomas for Calculating Standardized Incidence Ratios

This supplementary material has been provided by the authors to give readers additional information about their work.

© 2020 Gregersen PA et al. *JAMA Network Open.*

|eTable. Definition of sarcomas for calculating standardized incidence ratios

The classification of main cancer groups in the Danish Cancer Registry does not include a specific registration of sarcomas. Sarcoma cases were therefore identified by ICD-O-3 morphology codes (1978-2016) and ICD-7 codes (1943-1977).

List of ICD-O-3 morphology codes (C_MORFO3 in the Danish Cancer Registry) used to identify sarcoma cases during 1978-2016.

C_MORFO3	Text	C_MORFO3	Text
88003	Sarcoma, NOS	90403	Synovial sarcoma, NOS
91803	Osteosarcoma, NOS	90423	Synovial sarcoma, epithelioid cell
87113	Glomus tumor, malignant	90433	Synovial sarcoma, biphasic
88013	Spindle cell sarcoma	90443	Clear cell sarcoma,NOS (except of kidney 8964/3)
88033	Small cell sarcoma	91203	Hemangiosarcoma
88043	Epithelioid sarcoma	91303	Hemangioendothelioma, malignant
88053	Undifferentiated sarcoma	91333	Epithelioid hemangioendothelioma, malignant
88063	Desmoplastic small round cell tumor	91503	Hemangiopericytoma, malignant
88103	Fibrosarcoma, NOS	91703	Lymphangiosarcoma
88113	Fibromyxosarcoma	92313	Myxoid chondrosarcoma
88143	Infantile fibrosarcoma	94733	Primitive neuroectodermal tumor
88153	Solitary fibrous tumor, malignant	95403	Malignant peripheral nerve sheath tumor
88253	Low grade myofibroblastisk sarkom	95603	Neurilemmoma, malignant
88303	Fibrous histiocytoma, malignant	95813	Alveolar soft part sarcoma
88323	Dermatofibrosarcoma, NOS	91813	Chondroblastic osteosarcoma
88403	Myxosarcoma	91823	Fibroblastic osteosarcoma
88503	Liposarcoma, NOS	91833	Telangiectatic osteosarcoma
88513	Liposarcoma, well differentiated	91853	Small cell osteosarcoma
88523	Myxoid liposarcoma	91873	Intraosseous well differentiated osteosarcoma
88543	Pleomorphic liposarcoma	91923	Parosteal osteosarcoma
88553	Mixed type liposarcoma	91933	Periosteal osteosarcoma
88903	Leiomyosarcoma, NOS	92203	Chondrosarcoma, NOS
88913	Epithelioid leiomyosarcoma	92213	Juxtacortical chondrosarcoma
89013	Pleomorphic rhabdomyosarcoma, adult type	92403	Mesenchymal chondrosarcoma
89023	Mixed type rhabdomyosarcoma	92433	Dedifferentiated chondrosarcoma
89103	Embryonal rhabdomyosarcoma	92503	Giant cell tumor of bone, malignant
89123	Spindle cell rhabdomyosarcoma	92603	Ewing sarcoma
89203	Alveolar rhabdomyosarcoma	92613	Adamantinoma of long bones
89903	Mesenchymoma, malignant	93703	Chordoma, NOS

© 2020 Gregersen PA et al. *JAMA Network Open.*

List of ICD-7 codes used to identify sarcoma cases during 1943-1977.

ICD-7	Text	ICD-7	Text
1960	Bones of skull and face	8551	Biliary duct, other sarcomas
1961	Mandible	8553	Gallbladder, other sarcomas
1962	Vertebral column	8560	Liver,not spec.as prim.,o.sarc.
1963	Rib,stern.,clav.,ass.joints	8570	Pancreas, other sarcomas
1964	Long bones of up.limb,scapula	8580	Peritoneum, other sarcomas
1965	Short bones of upper limb	8590	Retroperitoneum, other sarcomas
1966	Pelvic bones	8600	Nasal cavity, other sarcomas
1967	Long bones of lower limb	8601	Ethmoid sinus, other sarcomas
1968	Short bones of lower limb	8602	Maxillary sinus,other sarcomas
1969	Bones	8610	Larynx, other sarcomas
1970	Connective tissue, head	8611	Glottis, Vocal cord, NOS., Sarcoma.
1975	Connective tissue, body	8621	Lung, other sarcomas
1976	Connect.tis.,up.limb &should.	8622	Pleura, other sarcomas
1977	Connect.tis.,lower limb & hip	8627	Lung,uncert.whet.primary,o.sarc.
1979	Connect.tis.,site unspecified	8630	Tumor of lung, other sarcomas
2961	Mandible, adamantinoma	8640	Mediastinum, other sarcomas
3996	Metast.sarc.,excl.lymph nodes	8700	Breast, other sarcomas
5965	Dermatofibrosarc./short bones limb CIS	8701	Breast, left, other sarcomas
8400	Lip, other sarcomas	8702	Breast, right, other sarcomas
8401	Lip, lower, other sarcomas	8710	Cervix, other sarcomas
8402	Lip, upper, other sarcomas	8720	Corpus uteri, other sarcomas
8410	Tongue, other sarcomas	8730	Uterus NOS, other sarcomas
8411	Tongue, base, other sarcomas	8740	Uterus,corp.col.,NOS,o.sarc.
8420	Salivary glands, other sarcomas	8750	Ovary, other sarcomas
8430	Mouth, other sarcomas	8755	Fallopian tube, other sarcomas
8440	Palate, other sarcomas	8760	Vulva, other sarcomas
8442	Buccal, other sarcomas	8761	Vagina, other sarcomas
8450	Tonsil, other sarcomas	8762	Labium majus, other sarcomas
8460	Nasopharynx, other sarcomas	8763	Clitoris, other sarcomas
8470	Hypopharynx, other sarcomas	8765	Vulva & vagina, other sarcomas
8480	Pharynx, NOS, other sarcomas	8770	Prostate gland, other sarcomas
8500	Esophagus, other sarcomas	8780	Testis,other sarc.,side unspec.
8510	Stomach,other sarcomas	8781	Testis, other sarcomas, left
8511	Stomach,fundus,cardia,oth.sarc.	8782	Testis, other sarcomas, right
8512	Stomach,corpus,other sarcomas	8790	Penis, other sarcomas
8513	Stomach,pylorus,other sarcomas	8791	Scrotum, other sarcomas
8520	Small intestine,other sarcomas	8792	Epidid.and sperm. cord,o.sarc.
8521	Duodenum,other sarcomas	8800	Kidney, other sarcomas
8522	Ileum & jejunum,other sarcomas	8802	Renal, pelvis, other sarcomas
8530	Colon,other sarcomas	8810	Urinary bladder, other sarcomas
8531	Colon,ransverse,other sarcomas	8817	Urethra, other sarcomas
8532	Colon,desc.& sigm.flex.,o.sarc.	8911	Skin, other sarcomas, eye
8533	Colon,sigm.& rectosigm.,o.sarc.	8912	Skin, other sarcomas, ear
8535	Colon, cecum, other sarcomas	8913	Skin, other sarcomas, face
8540	Rectum, other sarcomas	8914	Skin,other sarcomas,scalp
8549	Intest.tract,part unsp.,o.sarc.	8915	Skin,other sarcomas,body
8550	Liver, other sarcomas	8916	Skin,other sarcomas,upper limb

© 2020 Gregersen PA et al. *JAMA Network Open.*

List of ICD-7 codes used to identify sarcoma cases during 1943-1977 (continued).

ICD-7	Text	ICD-7	Text
8917	Skin,other sarcomas,lower limb	8940	Thyroid, other sarcomas
8919	Skin,other sarcomas,site unspec.	8950	Suprarenal gland, other sarc.
8920	Eye, other sarcomas	8954	Pituitary gland, other sarcomas
8923	Orbit, other sarcomas	8991	Unsp.org.head & neck,oth.sarc.
8930	Brain, other sarcomas	8992	Unsp.org.thorax,other sarcomas
8931	Spinal cord, other sarcomas	8993	Unsp.org.abdomen,other sarc.
8932	Meninges, other sarcomas	8994	Unsp.org.column & pelv.,o.sarc.
8933	P.nerv.,neurog.-neurofibrosarc.	8995	Unsp.org.extr.,other sarcomas
8934	P.nerves, other sarcomas	8999	Unsp.org.NOS,other sarcomas
8935	Spinal cord, neurofibrosarcoma		

© 2020 Gregersen PA et al. *JAMA Network Open.*
